# Application of a Hybrid Multi-Criterion Decision-Making Model for Evaluation and Improvement of Nurses' Job Satisfaction

**DOI:** 10.3389/fpubh.2022.896061

**Published:** 2022-07-04

**Authors:** Chao Liu, Huili Zhou, Yanjun Jin, Yen-Ching Chuang, Ching-Wen Chien, Tao-Hsin Tung

**Affiliations:** ^1^Institute for Hospital Management, Tsinghua University, Shenzhen, China; ^2^Taizhou Hospital of Zhejiang Province Affiliated With Wenzhou Medical University, Taizhou, China; ^3^Institute of Public Health and Emergency Management, Taizhou University, Taizhou, China; ^4^Business College, Taizhou University, Taizhou, China; ^5^Evidence-Based Medicine Center, Taizhou Hospital of Zhejiang Province Affiliated to Wenzhou Medical University, Linhai, China

**Keywords:** nurse job satisfaction, McCloskey/Mueller satisfaction scale (MMSS), Decision-Making Trial and Evaluation Laboratory (DEMATEL), Importance-Performance Analysis (IPA), multi-criterion decision-making (MCDM)

## Abstract

**Background:**

The global shortage and turnover of nurses is a current challenge. Past studies have shown that nurse job satisfaction may ameliorate nurse shortage. Although there are many studies on the criteria influencing nurses' job satisfaction, few have examined the causal relationships and weight of each criterion from a systematic perspective.

**Objective:**

Identify the key criteria and causal relationships that affect nurses' job satisfaction, and help nurse leaders identify high-weight, high-impact dimensions and contextualize them for improvement.

**Methods:**

The study developed a hybrid multi-criterion decision-making model, which incorporated the McCloskey/Mueller satisfaction 13-item scale (MMSS-13), and the Decision-Making Trial and Evaluation Laboratory and the Importance-Performance Analysis methods the model was used to analyze key factors of nurse satisfaction and their interrelationships based on the experience of 15 clinical nurse specialists.

**Results:**

In MMSS-13's dimension level, “satisfaction with work conditions and supervisor support” (*C*_5_) had the highest impact, and “satisfaction with salary and benefits” (*C*_1_) had the highest weight. In criteria level, “salary” (*C*_11_), “flexibility in scheduling time off” (*C*_24_), “maternity leave time” (*C*_31_), “opportunities for social contact after work” (*C*_41_), and “your head nurse or facility manager” (*C*_51_) had high influence under their corresponding dimensions. The “benefits package” (*C*_13_) was the top criterion with the highest impact on MMSS-13.

**Conclusions:**

This study assessed nurses' job satisfaction from a multidimensional perspective and revealed the causal relationships between the dimensions. It refined the assessment of nurse job satisfaction to help nurse leaders better assess nurse job satisfaction and make strategic improvements. The study found that compensation and benefits had the highest weight in nurses' job satisfaction. Meanwhile, support for family responsibilities and working conditions, and support from supervisors were the cause dimensions of job satisfaction. Among the more detailed criteria, salary, benefits package, maternity leave time, and leadership had a greater impact on nurses' job satisfaction. Nurse leaders should start with these dimensions to achieve efficient improvement of nurses' job satisfaction.

## Introduction

Nurses form the largest group of front-line healthcare workers fighting the COVID-19 pandemic (1). In view of the unprecedented efforts of nurses worldwide to contain and mitigate the pandemic, WHO designated 2020–21 as the International Year of Nurses and Midwives (2). Some scholars believe that the future of nursing will determine the fate of our health services, based on the contribution of nurses to COVID-19 (3). However, according to the State of the World's Nursing 2020 report, there was a global shortage of approximately 5.9 million nurses in 2018, and a predicted demand gap of 5.7 million nurses by 2030 (4). This shortage and turnover of nurses is a major issue in healthcare (5, 6).

Studies have shown that improving nurse job satisfaction can reduce nurse turnover and shortages (7, 8). Mahoney et al. found that nurse job satisfaction among nurse anesthetists in the United States (US) had a direct negative effect on the propensity to leave (9). Li et al. derived similar results while investigating the propensity of emergency nurses to leave and concluded that nurse job satisfaction should be improved to reduce the propensity (10). Qian et al. found a positive effect of job satisfaction on nurse retention intentions in a sample of nurses from several hospitals (11). Therefore, the evaluation and improvement of nurses' job satisfaction is an issue worth studying.

Improving nurses' job satisfaction involves multiple criteria and requires fine-grained management (12–14). Before implementing measures to improve nurses' job satisfaction, a thorough evaluation should be conducted. Knowing the current nurse job satisfaction in hospitals will help to take corresponding measures. Therefore, this study aimed to propose a method to systematically measure nurse job satisfaction from a managerial and applied perspective and to point out opportunities for improvement in nurse job satisfaction in hospitals.

We developed a hybrid multi-criterion decision-making (MCDM) model, which incorporated the McCloskey/Mueller satisfaction 13-item scale (MMSS-13), and the Decision-Making Trial and Evaluation Laboratory (DEMATEL) and Importance-Performance Analysis (IPA) methods. First, the MMSS-13 was used as an index system to measure nurses' job satisfaction. Second, the DEMATEL method was used as a systemic structure analysis technique for building an influential network-relation map (INRM) and influential weights for all criteria of the MMSS-13 index system. The decision-makers could understand several key criteria affecting nurses' job satisfaction from the system perspective. Lastly, the IPA method was used to analyze the nurses' job satisfaction for the case hospital, which obtained several key improvement criteria and corresponding management improvement strategies.

The main difference between this and other existing studies is the system-based assessment of nurses' job satisfaction through the DEMATEL-IPA approach. This study can help nurse leaders identify high-weighted and high-impact dimensions and improve them in the context of the actual situation. This leads to more efficient and effective improvements. The proposed hybrid MCDM model can help the nursing department propose more effective alternatives for improving the nurse job satisfaction environment.

The paper is organized as follows: Section Literature Review provides relevant literature on indicators and assessment methods for nurses' job satisfaction. Section Materials and Methods describes the MMSS-13, MCDM methods, and study design. Section Results describes the results of applying the hybrid MCDM model in the case hospital. Section Discussions further discusses the results of INRM and influential weights for all criteria, the improvement strategies of nurses' job satisfaction for case hospitals, as well as research limitations. Section Conclusion presents conclusions.

## Literature Review

Nurse job satisfaction evaluation is usually based on two steps: (1) to identify criteria that affect nurses' job satisfaction, and (2) to adopt an assessment method. Considering these two steps, this study reviewed the literature from two aspects: nurse job satisfaction criteria and evaluation methods.

### Criteria of Nurse Job Satisfaction in the Literature

Some studies have been conducted focusing on a single dimension for impact dimensions of nurses' job satisfaction. For example, Cortese et al. showed that work-family conflict reduces nurses' job satisfaction (15). Gottlieb et al. found that the degree of leadership empowerment affects nurses' autonomy and job agency, which in turn affects job satisfaction (16). Ho et al. showed that organizational support also influences nurses' job satisfaction (17). Dutra and Guirardello found that a good work environment increases nurses' job satisfaction (18). Hudgins et al. found a negative relationship between toxic leadership and employee satisfaction (19).

Due to the lack of a systematic perspective on assessing nurses' job satisfaction in this type of study, some scholars have developed nurse job satisfaction scales from multiple levels. For instance, the MMSS-13 is used to assess clinical nurse satisfaction (20), and Home Healthcare Nurses' Job Satisfaction Scale is designed to measure the job satisfaction of community nurses (21). A systematic review article suggests that the greatest requirement for research is the relative importance and causality of criteria that influence nurses' job satisfaction (22). The lack of research in this area has prevented managers from developing effective management practices, which has hindered the development of nurse retention management (22). After a careful review of the literature, this study adopted MMSS-13 as the assessment system because it is more widely used and validates and contains most of the dimensions mentioned in the literature.

### Nurse Job Satisfaction Assessment Methods in the Literature

After identifying nurse job satisfaction dimensions, the second stage required the selection of an appropriate method to integrate and rank the dimensions. According to current literature, surveys are used in most studies. For example, investigating job satisfaction and burnout of pediatric nurses in Turkey, Kaya et al. concluded that pediatric nurse managers should support younger and inexperienced nurses, thereby increasing their job satisfaction (23). Serafin et al. conducted a cross-sectional survey of nurses in Polish and Swedish surgical wards and concluded that the possibility of achievement, development of professional skills, and promotion were important factors influencing job satisfaction (24). Yasin et al. conducted a cross-sectional survey of job satisfaction factors among Ontario nurses and found that job satisfaction was related to factors such as peer support and work conditions, and suggested that satisfaction could be improved by moderating these specific factors (25). In conclusion, existing studies either assess nurses' job satisfaction in terms of a single dimension or analyze multiple dimensions, but ignore the relative importance and relationships between dimensions (26).

Therefore, a multi-perspective, in-depth assessment tool is required. MCDM is a modern scientific decision-making method for evaluating and improving multidimensional systems, widely used in various fields, such as strategic management of companies (27), evaluation of new technologies (28), and agricultural production (29). It is also used in the medical field. Alossta et al. applied the AHP-RAFSI approach to emergency medical service siting planning and found that the road network was the best location to deploy ambulances (30). Hsu et al. used Z-DEMATEL to rank trends in Taiwan's healthcare industry and found that the Internet of Things (IoT) and telemedicine are important trends for future development (31). Biswass' comparative analysis of the supply chain performance of leading Indian healthcare organizations using three different MCDM frameworks found that large-cap companies do not necessarily perform well (32). Mardani et al. reviewed the application of MCDM in health care and medical problems from 1989 to 2018, and the results showed that analytic hierarchy process (AHP) techniques and mixed methods were the most commonly used in health care (33). MCDM techniques have a remarkable ability to solve and assess existing problems in the health care setting (33).

Analytic hierarchy process and DEMATEL are some commonly used MCDM techniques for assessing job satisfaction. For instance, Bonyadi et al. used the AHP method to analyze employee satisfaction in water companies (34). Tsai used DEMATEL to explore the job satisfaction evaluation criteria of R&D personnel (35). Xin et al. used the DEMATEL method to find that salary, future development, workload, time occupation, and supervisory stress were the main factors affecting the job satisfaction of pharmacy employees (36).

The above literature analysis shows that MCDM methods have been widely used in healthcare to address multidimensional issues. Meanwhile, the more commonly used methods for job satisfaction evaluation are questionnaire-based analysis, AHP, and DEMATEL. In the field of nurses' job satisfaction assessment, there is a lack of studies using MCDM for analysis. Considering that AHP cannot deal with the interrelationship of dimensions of nurses' job satisfaction, this study systematically assessed the weights and interactions of each dimension by using the DEMATEL method. Additionally, IPA was also used in this study to analyze the case hospital and present specific suggestions.

## Materials and Methods

### The MMSS-13 Scale

The MMSS was developed by Mueller and McCloskey for measuring nurse job satisfaction in 1974 (37). They later revised the original MMSS evaluation system and formed the MMSS-31 (20). The MMSS-31 has high reliability for measuring nurses' job satisfaction (Cronbach's alpha is 0.89) (20). The MMSS-31 is used for job satisfaction of different types of nurses, such as ambulatory (38), home (39), and long-term care (40). However, Abu Ajamieh et al. concluded that MMSS-31 needs to be modified to improve reliability for applications outside the USA (41). Some scholars have proposed different modified versions of the MMSS-31 concerning different cultures, such as the Kuwait version (MMSS-21) and the Canadian version (MMSS-23) (42, 43). Clinton et al. found that up to 45% of items may be redundant based on the environment and context in which the MMSS-31 is used, and proposed the MMSS-13, comprising 13 core items (44). The MMSS-13 has better psychometric characteristics and good internal consistency (Cronbach's alpha is .82) (44). Clinton et al. mentioned that for complex causal analysis studies, the most effective approach is to combine the MMSS-13 with specific items (44). The items in MMSS-13 are more central, which better meets the requirements of this study to identify key criteria, and causal relationships, and propose effective management strategies. The evaluation criteria of the MMSS-13 scale are shown in Table 1.

**Table 1:** The MMSS-13 scale (Cronbach's Alpha = 0.82).

**Dimension**	**Criteria**
Satisfaction with salary and benefits (*C*_1_)	Salary (*C*_11_)
	Vacation (*C*_12_)
	Benefits package (*C*_13_)
Satisfaction with scheduling (*C*_2_)	Flexibility in scheduling your hours (*C*_21_)
	Opportunity to work consecutive days (*C*_22_)
	Weekends off per month (*C*_23_)
	Flexibility in scheduling time off (*C*_24_)
Satisfaction with support for family responsibilities (*C*_3_)	Maternity leave time (*C*_31_)
	Child care for employees' children at facility (*C*_32_)
Satisfaction with social and interaction opportunities (*C*_4_)	Opportunities for social contact after work (*C*_41_)
	Professional interactions with other disciplines (*C*_42_)
Satisfaction with work conditions and supervisor support (*C*_5_)	Your head nurse or facility manager (*C*_51_)
	Decision making (*C*_52_)

### The DEMATEL Method

A systematic structural approach was proposed by the Battelle Memorial Institute to solve complex decision-making problems in real life (45). This method and Interpretative Structural Modeling belong to the classic methods of multi-criteria decision-making methodology for identifying the interrelated structure between criteria (46). First, this method constructs the degree of interaction between criteria from the practical experience of domain experts. Second, through a series of matrix operations, the influence properties of all criteria and the influence network diagram are obtained. This method is widely used in various thematic areas, such as urban resilience (47), environmental sustainability innovation (48), and public health (49).

There were interactive relationships among the dimensions of nurses' job satisfaction in this study, and identifying causal relationships can help managers develop key causal strategies. Compared to methods that only determine factor weights, such as AHP (50) and best-worst method (BWM) (51), DEMATEL is advantageous as it can deal with the causal relationships between criteria, and further identify key criteria from network relationships (52). Garg used the advantages of DEMATEL to identify the interdependencies between e-waste reduction strategies, thus helping managers to develop key causal strategies (53). Badri Ahmadi et al. used the Z-DEMATEL technique to analyze the interactions and interdependencies among innovation factors for environmental sustainability (48). Although ANP can also analyze the grid relationship between dimensions (54), DEMATEL's influence network diagram is more conducive for managers to adopt strategies. Regarding the calculation of the method, this paper refers to the study of Chuang et al. (55, 56). The brief calculation steps are described as follows:

Step 1: Establish an initial direct impact relationship matrix (***S***) from the clinical experience of (*k*) nurses. First, based on the MMSS-13 scale with (*n*) criteria, nurses assessed the degree of interaction between criterion (*i*) and criterion (*j*) based on a five-point Likert scale [i.e., from no impact (0) to very high impact (4)]. Then, they established a direct influence relationship matrix (***C*** = [*c*_*ij*_]_*n* × *n*_) from their own clinical experience. Finally, the (*k*) direct influence relationship matrices (***C*** = [*c*_*ij*_]_*n* × *n*_) were averaged to create an average direct influence relationship matrix [i.e., the initial direct impact relationship matrix (***S***)], which represents the clinical experience of the respondents, as shown in Equation (1).
  (1) $$\begin{array}{r} S=\left[\begin{array}{ccccc} s_{11} & \cdots & s_{1j} & \cdots & s_{1n}\\ \vdots & \ddots & \vdots & \ddots & \vdots \\ s_{i1} & \cdots & s_{ij} & \cdots & s_{in}\\ \vdots & \ddots & \vdots & \ddots & \vdots \\ s_{n1} & \cdots & s_{nj} & \cdots & s_{nn} \end{array}\right]={\left[\left(\sum_{\varphi =1}^{k}c_{ij}^{\varphi }\right)/k\right]}_{n\times n} \end{array}$$
Step 2: Convert the initial direct impact relationship matrix (***S***) into a regularized influence relation matrix *N* in proportional form as shown in Equations (2) and (3).
  (2) $$\begin{array}{r} \Omega =\max\left\{\max\sum_{j=1}^{n}s_{ij},\max\sum_{i=1}^{n}s_{ij}\right\},i,j\in \left\{1,2,...,n\right\} \end{array}$$
  (3) $$\begin{array}{r} N=\frac{S}{\Omega } \end{array}$$
Step 3: Derive the integrated influence relationship matrix (***R***) from the regularized influence relationship matrix (***N***) through Equation (4), where *I* is the unit matrix.
  (4) $$\begin{array}{l} R=N+N^{2}+...+N^{\Phi }=N{\left(I-N\right)}^{-1},\\ \text{when }\underset{\Phi \to \infty }{\lim}N^{\Phi }={\left[0\right]}_{n\times n} \end{array}$$
Step 4: Construct impact indicators for the criteria from the integrated impact relationship matrix (*R*). The influence indicator (*d*_*i*_) is the row vector sum of the matrix (*R*), which represents the total degree of influence of the criterion (*i*) on other criteria (Equation 5). The affected indicator (*u*_*i*_) is the column vector sum of the matrix (*R*), which represents the total degree of influence of criterion (*i*) by other criteria (Equation 6).

(5) $$\begin{array}{r} \text{Influence indicator: }d_{i}\;=\left(d_{1},d_{2},\ldots ,d_{n}\right)={\left[\sum_{j=1}^{n}r_{ij}\right]}_{n\times 1}^{} \end{array}$$

(6) $$\begin{array}{l} \text{Affected indicator: }u_{i}\;=\left(u_{1},u_{2},\ldots ,u_{n}\right)={\left(u_{j}\right)}_{1\times n}^{T}\\ \;={\left[\sum_{i=1}^{n}r_{ij}\right]}_{1\times n}^{T} \end{array}$$

Where, *T* is the transpose.

The other two composite indicators are the centrality indicator (*d*_*i*_ + *u*_*i*_) and the causality indicator (*d*_*i*_ − *u*_*i*_). The centrality indicator (*d*_*i*_ + *u*_*i*_) indicates the strength of association of the criterion's influence (*i*) within the whole evaluation system. The causality indicator (*d*_*i*_ − *u*_*i*_) indicates the influence property of the criterion (*i*) itself within the whole evaluation system, and thus all criteria can be divided into a group of causes [i.e., *d*_*i*_ − *u*_*i*_ > 0, which indicates that criterion (*i*) is biased toward the cause nature] and a group of effects [i.e., *d*_*i*_ − *u*_*i*_ < 0, which indicates that criterion (*i*) is biased toward the effect nature].

Step 5: Construct the INRM of criteria within the evaluation system. The space of this diagram is constructed by the centrality (*d*_*i*_ + *u*_*i*_) and causality (*d*_*i*_ − *u*_*i*_) indicators. Based on the INRM, the director of nursing can visualize the structure of interinfluence relationships among all criteria.
Step 6: Define the influential weight of the criterion from the centrality indicator (*d*_*i*_ + *u*_*i*_). The weight represents the ratio of correlation strength between the criterion (*i*) and other criteria in the whole evaluation system for the evaluation object, as shown in Equation (7).
  (7) $$\begin{array}{r} w_{i}=\frac{d_{i}+u_{i}}{\sum_{i=1}^{n}\left(d_{i}+u_{i}\right)} \end{array}$$

### IPA Method

**Figure 1:**
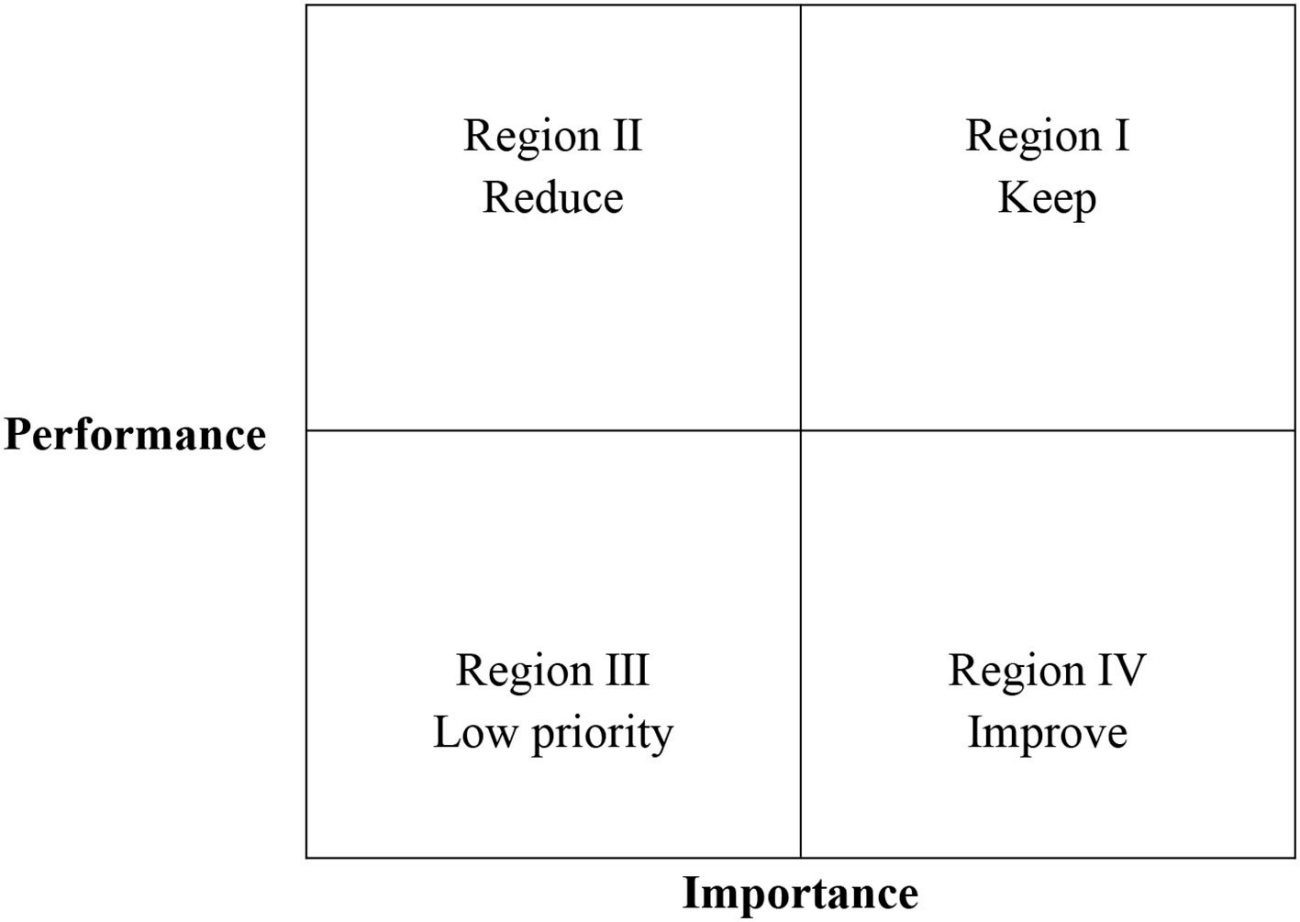
IPA matrix.

Importance-Performance Analysis was proposed by Marilla and James for providing an easy analytical tool for service quality analysis and improvement (57). It integrates the importance and performance of criteria into a four-quadrant diagram, as shown in Figure 1. The method is an easy-to-understand management insight and decision-making direction for criteria or factors in a four-quadrant diagram. IPA is commonly used for satisfaction evaluation; the model is intuitive and clear (58, 59), which facilitates nurse managers to quickly understand the current situation and make improvements. Until now, the method has been applied in different areas, such as higher education (60), service quality (61), and information systems (62). The application steps of the method in this study are as follows (63):

Step 1: Based on MMSS-13, nursing specialists evaluated the satisfaction performance of all criteria by using a five-point Likert scale [i.e., from very dissatisfied (1) to very satisfied (5)].
Step 2: The weight of DEMATEL and the satisfaction of the case hospital were the x- and y-axes of the IPA diagram, respectively.
Step 3: Based on the IPA diagram, all criteria could be distinguished into four regions, which were different characteristics and corresponding management methods: (I) Keep: decision-makers should continue to invest resources to maintain current satisfactory performance. (II) Reduce: decision-makers should reduce the input of resources to avoid waste or inefficient use of resources. (III) Low priority: decision-makers should consider not investing resources to improve the satisfaction performance of these indicators for the time being. (IV) Improve: decision-makers should invest resources to improve current satisfactory performance.

### Study Design and Data Collection

Information about the participants in this study was collected anonymously. All procedures were performed in accordance with the guidelines of the Ethics Committee of Zhejiang Taizhou Hospital (Approval Number: K20220235) and the tenets of the Declaration of Helsinki. The questionnaire consisted of two parts, both using a five-point Likert scale. The first part was the DEMATEL questionnaire and the second part was a self-assessment form on nurses' job satisfaction. The questionnaire was distributed to 15 nursing specialists with extensive clinical work experience and was conducted in March 2022. Nursing specialists were predominantly supervisor nurses (46.7%). In addition, most of the specialists had a bachelor's degree (46.7%) and had 15 years or more of nursing experience (53.4%). Details of the specialists are shown in Table 2.

**Table 2:** The background description of 15 nursing specialist.

**Characteristics**	**Value (%)**
**Gender**	
Male	0 (0%)
Female	15 (100%)
**Age**	
<30	5 (33.3%)
30–39	8 (53.3%)
≥40	2 (13.4%)
**Education**	
Associate Degree	3 (20%)
Bachelor	7 (46.7%)
Master or above	5 (33.3%)
**Years of service**	
Under 10 years	5 (33.3%)
10–14	2 (13.3%)
15 and above	8 (53.4%)
**Professional title**	
Senior nurse	5 (33.3%)
Supervisor Nurse	7 (46.7%)
Chief Nurse	3 (20%)

## Results

### DEMATEL Results for Composite Indicators and Weights

The initial direct impact relationship matrix (*S*) (Table 3) was obtained from the questionnaire of 15 nursing experts. The statistical significance of the matrix (*S*) was 98.05%, and the gap error was 1.95% with a good confidence level. The results of centrality (*d*_*i*_ + *u*_*i*_) and causality (*d*_*i*_ − *u*_*i*_) indicators for dimensions and corresponding criteria are shown in Table 4.

**Table 3:** The initial direct impact relationship matrix *S*.

	***C*_11_**	***C*_12_**	***C*_13_**	***C*_21_**	***C*_22_**	***C*_23_**	***C*_24_**	***C*_31_**	***C*_32_**	***C*_41_**	***C*_42_**	***C*_51_**	***C*_52_**
*C*_11_	0.000	2.733	3.000	2.467	2.467	2.400	2.333	2.400	2.333	2.200	2.133	2.200	2.467
*C*_12_	2.600	0.000	2.733	2.467	2.200	2.467	2.667	2.733	2.533	2.400	2.400	2.133	2.133
*C*_13_	2.933	3.067	0.000	3.267	2.733	2.600	2.333	3.200	3.200	2.267	2.067	2.000	2.000
*C*_21_	2.200	2.600	2.200	0.000	2.400	2.333	2.267	2.400	2.200	3.067	2.800	2.133	2.267
*C*_22_	2.267	2.533	2.467	2.600	0.000	2.533	2.667	2.733	2.067	2.200	2.933	2.000	2.000
*C*_23_	2.333	2.333	2.400	2.933	2.733	0.000	2.600	2.333	2.467	2.133	2.467	2.267	2.000
*C*_24_	2.200	2.733	2.600	2.600	2.800	2.733	0.000	2.533	2.267	3.133	2.933	2.333	1.933
*C*_31_	2.800	3.600	3.400	2.867	2.733	3.067	2.267	0.000	2.200	2.400	2.133	2.267	2.133
*C*_32_	2.000	2.333	2.267	2.333	2.267	2.333	2.200	2.400	0.000	2.200	2.333	2.200	2.067
*C*_41_	2.067	2.400	2.400	2.400	2.133	2.467	2.400	2.533	2.467	0.000	2.267	2.267	2.200
*C*_42_	1.800	1.800	2.600	2.133	2.067	2.067	2.200	2.133	2.067	2.267	0.000	2.267	2.200
*C*_51_	2.133	2.267	2.400	2.533	2.467	2.467	2.467	2.333	2.333	2.333	2.333	0.000	2.533
*C*_52_	2.000	2.000	2.133	2.200	2.200	2.133	2.267	2.133	2.267	2.267	2.267	2.200	0.000

*The significant confidence equation is $\left(\left(\sum_{i=1}^{n}\sum_{j=1}^{n}|s_{ij}^{15}-s_{ij}^{15-1}|/s_{ij}^{15}\right)/\left(n\left(n-1\right)\right)\right)\times 100\%=1.95\%<5\%$, i.e., significant confidence is 98.05%*.

**Table 4:** The results of impact indicator for dimensions and criteria.

	***d*_*i*_**	***u*_*i*_**	***d*_*i*_+*u*_*i*_**	***d*_*i*_-*u*_*i*_**	**Group**
Satisfaction with salary and benefits (*C*_1_)	3.859	3.778	7.638	0.081	Cause
Salary (*C*_11_)	9.794	9.236	19.030	0.559	Cause
Vacation (*C*_12_)	9.901	10.198	20.098	−0.297	Effect
Benefits package (*C*_13_)	10.595	10.237	20.832	0.358	Cause
Satisfaction with scheduling (*C*_2_)	3.777	3.793	7.570	−0.017	Effect
Flexibility in scheduling your hours (*C*_21_)	9.669	10.313	19.981	−0.644	Effect
Opportunity to work consecutive days (*C*_22_)	9.746	9.799	19.545	−0.053	Effect
Weekends off per month (*C*_23_)	9.737	9.928	19.666	−0.191	Effect
Flexibility in scheduling time off (*C*_24_)	10.293	9.627	19.920	0.665	Cause
Satisfaction with support for family responsibilities (*C*_3_)	3.763	3.728	7.490	0.035	Cause
Maternity leave time (*C*_31_)	10.674	10.023	20.697	0.650	Cause
Child care for employees' children at facility (*C*_32_)	9.086	9.554	18.639	−0.468	Effect
Satisfaction with social and interaction opportunities (*C*_4_)	3.451	3.701	7.152	−0.250	Effect
Opportunities for social contact after work (*C*_41_)	9.425	9.700	19.125	−0.274	Effect
Professional interactions with other disciplines (*C*_42_)	8.666	9.757	18.423	−1.091	Effect
Satisfaction with work conditions and supervisor support (*C*_5_)	3.509	3.358	6.866	0.151	Cause
Your head nurse or facility manager (*C*_51_)	9.600	8.864	18.464	0.736	Cause
Decision making (*C*_52_)	8.798	8.748	17.546	0.050	Cause

The results of causality indicator (*d*_*i*_ − *u*_*i*_) analysis for dimension level, “Satisfaction with salary and benefits” (*C*_1_), “Satisfaction with support for family responsibilities” (*C*_3_), and “Satisfaction with work conditions and supervisor support” (*C*_5_) were the cause indicators, which were the dimensions that mainly affected the other dimensions in MMSS-13. “Satisfaction with scheduling” (*C*_2_) and “Satisfaction with social and interaction opportunities” (*C*_4_) was the effect indicators, which were the dimensions that were mainly affected by the other dimensions in MMSS-13. The ranking results of the centrality indicator (*d*_*i*_ + *u*_*i*_) from high to low were “Satisfaction with salary and benefits” (*C*_1_), “Satisfaction with support for family responsibilities” (*C*_3_), and “Satisfaction with work conditions and supervisor support” (*C*_5_) would affect “Satisfaction with scheduling” (*C*_2_) and “Satisfaction with social and interaction opportunities” (*C*_4_).

The influential weight of the criteria is shown in Table 5. “Satisfaction with salary and benefits” (*C*_1_) had the highest weight in the dimension level. For the criteria level, “Benefits package” (*C*_13_), “Maternity leave time” (*C*_31_), and “Vacation” (*C*_12_) were the top three highest weights.

**Table 5:** The results of influential weights for dimensions and criteria.

**Dimensions**	**Local weight**	**Ranking**	**Criteria**	**Local weight**	**Ranking**	**Global weight**	**Ranking**
*C*_1_	0.208	1	*C*_11_	0.317	3	0.076	9
			*C*_12_	0.335	2	0.080	3
			*C*_13_	0.347	1	0.083	1
*C*_2_	0.206	2	*C*_21_	0.253	1	0.079	4
			*C*_22_	0.247	4	0.078	7
			*C*_23_	0.249	3	0.078	6
			*C*_24_	0.252	2	0.079	5
*C*_3_	0.204	3	*C*_31_	0.526	1	0.082	2
			*C*_32_	0.474	2	0.074	10
*C*_4_	0.195	4	*C*_41_	0.509	1	0.076	8
			*C*_42_	0.491	2	0.073	12
*C*_5_	0.187	5	*C*_51_	0.513	1	0.073	11
			*C*_52_	0.487	2	0.070	13

### IPA Results of Four Regions for Case Hospital

The IPA results as shown in Table 6 and Figure 3. “Opportunity to work consecutive days” (*C*_22_), “Weekends off per month” (*C*_23_), “Flexibility in scheduling time off” (*C*_24_), and “Maternity The leave time” (*C*_31_) were included in the (I) Keep region; “Opportunities for social contact after work” (*C*_41_), “Professional interactions with other disciplines” (*C*_42_), “Your head nurse or facility manager” (*C*_51_), “Decision making” (*C*_52_) in the (II) Reduce region; “Salary” (*C*_11_) and “Child care for employees' children at the facility” (*C*_32_) in the (III) Low Priority region; and “Vacation” (*C*_12_), “Benefits package” (*C*_13_), and “Flexibility in scheduling your hours” (*C*_21_) in the (IV) Improve region.

**Table 6:** IPA results.

**Indicators**	**Importance**	**Performance**	**Region**
Salary (*C*_11_)	0.076	3.000	III (Low priority)
Vacation (*C*_12_)	0.080	3.067	IV (Improve)
Benefits package (*C*_13_)	0.083	2.933	IV (Improve)
Flexibility in scheduling your hours (*C*_21_)	0.079	3.333	IV (Improve)
Opportunity to work consecutive days (*C*_22_)	0.078	3.600	I (Keep)
Weekends off per month (*C*_23_)	0.078	3.533	I (Keep)
Flexibility in scheduling time off (*C*_24_)	0.079	3.733	I (Keep)
Maternity leave time (*C*_31_)	0.082	3.533	I (Keep)
Child care for employees' children at facility (*C*_32_)	0.074	2.733	III (Low priority)
Opportunities for social contact after work (*C*_41_)	0.076	3.733	II (Reduce)
Professional interactions with other disciplines (*C*_42_)	0.073	3.600	II (Reduce)
Your head nurse or facility manager (*C*_51_)	0.073	3.933	II (Reduce)
Decision making (*C*_52_)	0.070	3.400	II (Reduce)
Mean	0.077	3.395	

## Discussions

The DEMATEL and IPA results were integrated and analyzed to propose practical management measures.

### INRM and Influential Weight Analysis

**Figure 2:**
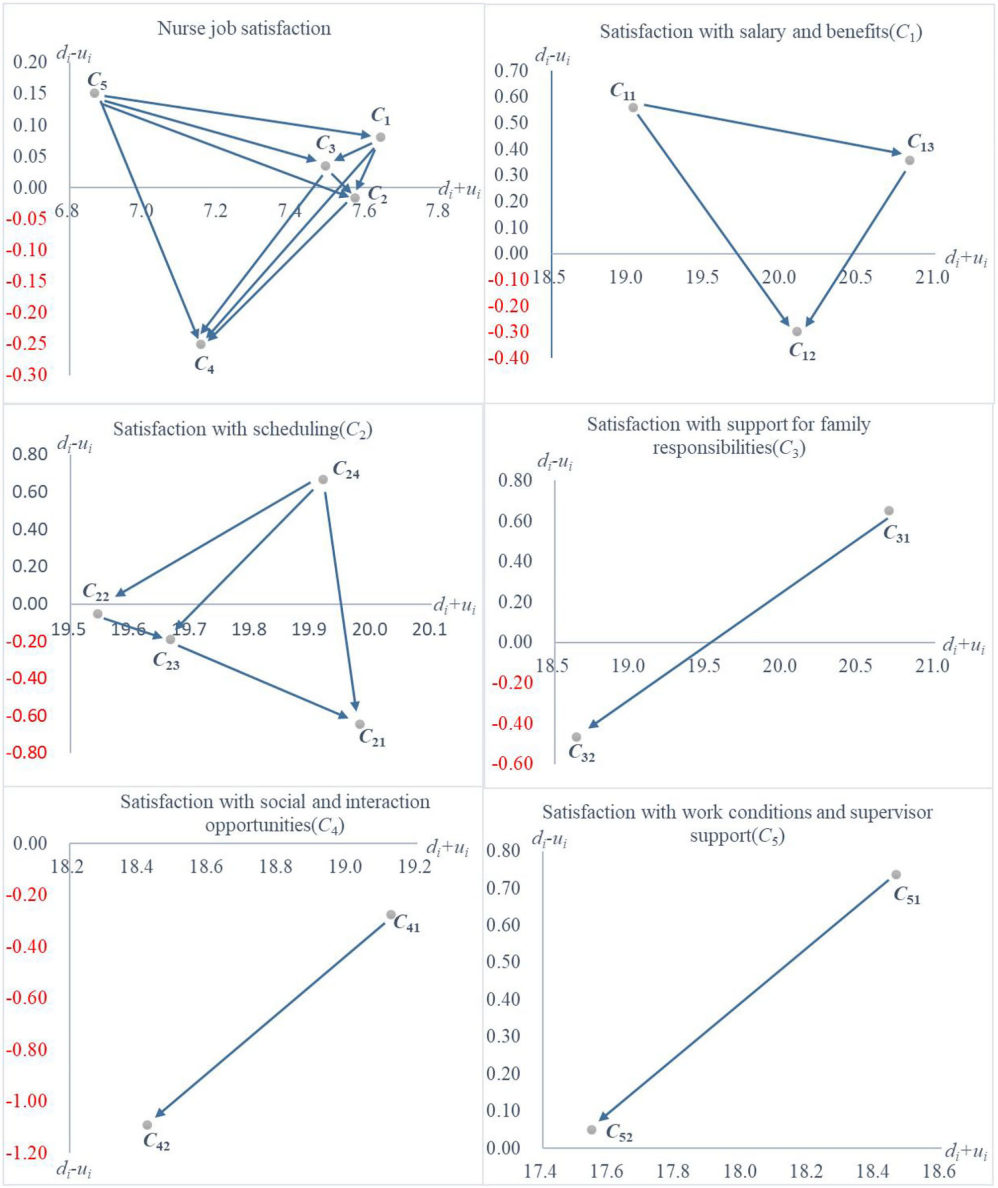
Influential network-relation map (INRM).

The INRM (Figure 2) illustrated the interdependent relationships between dimensions and criteria.

At the dimension level, “Satisfaction with work conditions and supervisor support” (*C*_5_) had a very strong influence on the satisfaction of other dimensions. Ravangard et al. showed that supervisor support had a significant negative effect on work-family conflict (64). Several past studies have also shown that improved working conditions and supervisor support can increase nurses' job satisfaction (65–67). In addition, “Satisfaction with salary and benefits” (*C*_1_) had the highest importance in nurses' job satisfaction. As some studies have shown, improved compensation and benefits can increase nurse satisfaction (12, 68–70).

Therefore, “Satisfaction with work conditions and supervisor support” (*C*_5_) had an overall effect on improving nurses' job satisfaction, while “Satisfaction with salary and benefits” (*C*_1_) was extremely important for it.

Regarding criteria level, “Salary” (*C*_11_) had the highest impact, and “Benefits package” (*C*_13_) had the greatest weight in “Satisfaction with salary and benefits” (*C*_1_). This may be partly due to the attractiveness of the nurse position reflected in its high salary and welfare. Simultaneously, there is a distinction between established and non-established nursing positions in China. Both the establishment and the title of the nurse affect the level of salary and benefits package (71). Misfeldt et al. found that while compensation and benefits were the most recognized methods to improve employee satisfaction and retention, improved work environment, flexible work hours, and family work balance also increased job satisfaction (72). “Flexibility in scheduling time off” (*C*_24_) had a high influence and weight under their corresponding dimensions. This may be because nurses are busy and predominantly female, and Chinese women play the role of caregivers in most families. Therefore, nurses have a greater need for freedom and flexibility. Finally, due to the high-power distance culture in China, “Your head nurse or facility manager” (*C*_51_) had a high impact on “Satisfaction with work conditions and supervisor support” (*C*_5_).

Therefore, “Satisfaction with salary and benefits” (*C*_1_), “Satisfaction with support for family responsibilities” (*C*_3_), and “Satisfaction with work conditions and supervisor support” (*C*_5_) should be focused on for improving nurses' job satisfaction. For example, California has improved nurse job satisfaction through salary improvements, increased RN staffing, and comprehensive leadership programs for nurses (73). Furthermore, the improvement of nurses' job satisfaction was closely related to the satisfaction of “Salary” (*C*_11_), “Benefits package” (*C*_13_), “Maternity leaves time” (*C*_31_), and “Your head nurse or facility manager” (*C*_51_).

### Improvement Strategies for the Case Hospital

**Figure 3:**
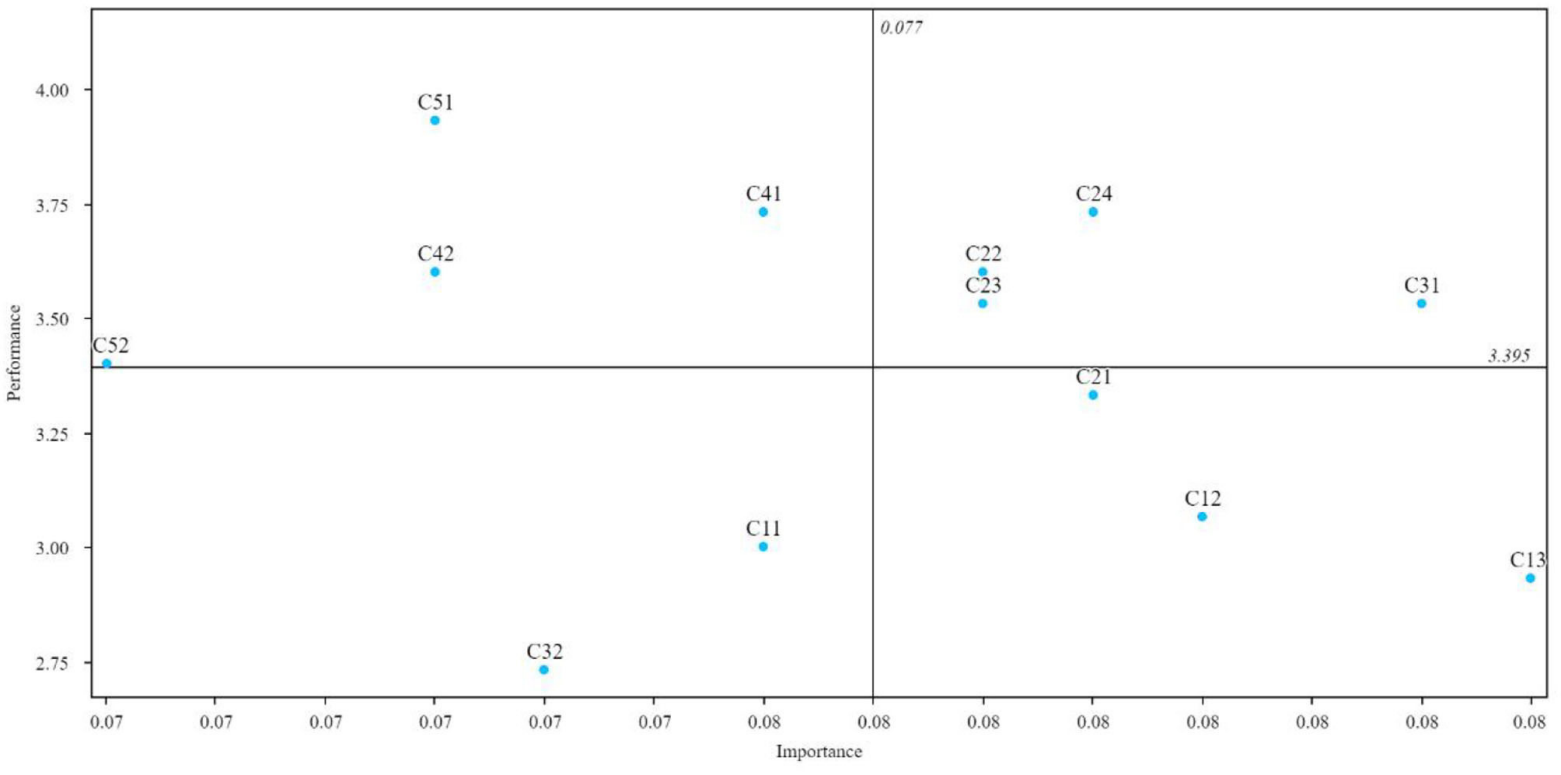
The IPA diagram of nurses' satisfaction.

Since the weights of DEMATEL were used in IPA, the analysis of DEMATEL results should focus on their causality in the integrated analysis. Based on Figures 2, 3, the “Benefits package” (*C*_13_) had high impact and weight, but low performance. Therefore, nursing directors of this hospital should focus “Benefits package” (*C*_13_) for improving nurses' job satisfaction. Improved compensation can increase satisfaction, but wage increases alone have a limited effect (74). “Vacation” (*C*_12_) and “Flexibility in scheduling your hours” (*C*_21_) were in the (IV) Improve region in IPA; therefore, nurse leaders should work upon these two dimensions as well, for example, by improving leave and work schedule flexibility through increased nursing staffing (73). Simultaneously, “Vacation” (*C*_12_) and “Flexibility in scheduling your hours” (*C*_21_) were both in the DEMATEL effect group. “Vacation” (*C*_12_) and “Flexibility in scheduling your hours” (*C*_21_) had a greater impact on “Vacation” (*C*_12_). The nursing director could coordinate the three in accordance with this relationship, for example, by improving the pay and benefits system for overtime (75). “Flexibility in scheduling your hours” (*C*_21_) was greatly affected by “Flexibility in scheduling time off” (*C*_24_), meaning that showing flexibility when nurses ask for time off work will be responded with enthusiasm afterward (75). “Your head nurse or facility manager” (*C*_51_) was in (II) Reduce region but had a high degree of cause and therefore measures should be taken to maintain it, such as improving job satisfaction through leadership and communication skills training (76).

In summary, the following points need to be noted to improve the job satisfaction of nurses in this hospital:

Nursing directors need to focus on improving nurse satisfaction with improved salaries and benefits packages. Further interviews and surveys revealed that nurses were not satisfied with their compensation package because of a significant gap compared to physicians. We recommended that the hospital's human resources department study and evaluate this gap.

Nursing directors should also pay attention to improving vacation, flexibility in scheduling hours, and flexibility in scheduling time off. Opportunities for social contact after work, professional interactions with other disciplines, and decision-making should be appropriately saved resources. Based on further interviews, some nurses felt that the hospital had so many social and learning activities that there was no private time or flexibility. We recommended that hospital administrators re-examine the necessity and autonomy of existing social and learning exchanges.

Opportunities to work on consecutive days, weekends off per month, flexibility in scheduling time off, maternity leave time, and head nurse or facility manager should continue to maintain these indicators. Child care for employees' children at the facility was not a priority indicator in the case hospital.

### Representativeness and Limitations of the Study

Nurse job satisfaction involves multiple criteria (12–14), and its improvement requires a multidimensional approach to understand the current situation and assess it through a comprehensive system. This study provided this multidimensional perspective and obtained the interrelationship between dimensions. Based on the experience and knowledge of nursing experts, the study obtained weight rankings and causal relationships for the dimensions of nurses' job satisfaction. Weight ranking helped nurse leaders identify key dimensions of job satisfaction. Causal relationships helped nurse leaders develop key causal strategies for improving job satisfaction. Moreover, the IPA methodology used in this study could also help nurse leaders visualize the current state of affairs and rationalize the allocation of improvement resources. Practitioners using this approach can assess their nursing staff and can then follow the steps outlined in this study to quickly derive IPA results for improvement.

The study of the DEMATEL method used in this study is dynamic; research on this method is increasing and can be applied to a variety of topics (77). It should be noted that the DEMATEL-IPA method assumes that all dimensions of a decision are interacting with each other and is not applicable to decisions where the dimensions are independent of each other. The classical DEMATEL method was used in this study, which is applicable to simple systems but not to decision-related problems with many dimensions and ambiguous environments. However, for such environments, it is also possible to integrate ambiguous set approaches to study, such as ambiguous DEMATEL (78), Z-DEMATEL (31), etc. Some scholars propose hierarchical DEMATEL methods to solve complex system problems with many system factors and influencing factors, and the existence of the hierarchy (79). There is a wide variety of MCDM methods, and different methods may produce different outputs, requiring the selection of an appropriate technique depending on the research question (33).

One of the limitations of this study is that the questionnaire structure was designed specifically for the nurses. However, using the corresponding questionnaire system, it is possible to apply it to other occupational groups (such as physicians) following the methodological steps of the study. Another limitation is that the results and decisions of this study were based on the clinical experience of 15 nursing specialists in the case hospitals. The results may vary across hospitals. The rigor and robustness of the assessment structure could be improved with the addition of feedback from different hospitals. Furthermore, the study found that past literature rarely applied MCDM in the area of nurse job satisfaction. Due to the uniqueness of the MCDM, it is difficult for us to use the available data for other methods (e.g., AHP, ANP). Therefore, it is difficult to conduct a more detailed comparative analysis with the results of past studies or other MCDM methods. This study serves as a beginning, and we expect more multi-criterion decision analysis methods to be applied to this topic in the future. We believe that the comparative analysis will be richer and more appropriate by then.

## Conclusion

The shortage of nurses has never been more pronounced than during the COVID-19 pandemic. The shortage and turnover of nurses have become a major problem in the healthcare system. Improving nurse job satisfaction can help alleviate this problem. A vital step in improving nurse job satisfaction is to assess and identify key factors. Many studies on nurse job satisfaction have focused on validating the relevance of a specific dimension or qualitatively listing multiple influencing dimensions. Few studies have assessed nurse job satisfaction with a multidimensional, weighted perspective. The interrelationships between the dimensions are also ignored. This study addresses this literature gap to help nurse leaders better assess nurse job satisfaction and make strategic improvements.

In this study, a multi-criterion decision model based on MMSS-13 was proposed. DEMATEL method was used to develop nurse job satisfaction criteria to help nursing department managers clarify the weights and causal relationships of each dimension. The DEMATEL results were used as the basis for visual analysis of the current situation of nurses' job satisfaction in the case hospital in combination with the IPA method. Then customized management improvement suggestions are proposed for the current situation of the case hospital.

The results of the analysis showed that salary and benefits remain one of the most important criteria for improving nurses' job satisfaction. Additionally, family responsibility, working conditions, and supervisor support are highly influential. These dimensions affect nurses' satisfaction with other aspects. Among the more detailed criteria, benefits package, maternity leave time, and leadership have a high impact on nurses' job satisfaction, as well as other dimensions. It is recommended that hospitals focus on assessing and improving related dimensions, such as compensation, optimization, and leadership training. Nursing managers can adopt a weighting system to conduct an IPA analysis of nurse satisfaction in their hospitals and take corresponding improvement measures based on the analysis results.

Assessing nurse job satisfaction with a multidimensional, causal perspective has the potential to increase the efficiency of nurse satisfaction improvement. The method has streamlined dimensions and can be applied to assess nurse job satisfaction on a large scale as a first step and guide to reducing nurse turnover in hospitals. Additionally, our questionnaire data could not be applied to other methods such as AHP and BWM. Therefore, it was difficult to comparatively analyze this study in detail against the results of past studies or other MCDM methods. Future research may include other appropriate MCDM methods. This method could be applied to medical staff's occupational health improvement and provide recommendations to managers. In addition, nurse job satisfaction assessment methods could be embedded in hospital information systems for ongoing assessment. Finally, studies can be conducted for different groups of nurses, such as home-based work nurses.

## Data Availability Statement

The raw data supporting the conclusions of this article will be made available by the authors, without undue reservation.

## Ethics Statement

The studies involving human participants were reviewed and approved by Institutional Review Board of Taizhou Hospital of Zhejiang Province, Affiliated With Wenzhou Medical University (Approval Number: K20220235). The patients/participants provided their written informed consent to participate in this study.

## Author Contributions

CL and Y-CC conducted the study and drafted the manuscript. HZ and YJ participated in the design and data collection of the study. CL calculated the results of this study and drew the influence network relation map. T-HT and C-WC conceived the study and participated in its design and coordination. All authors read and approved the final manuscript.

## Funding

This study was funded by the Specialized Subsidy Scheme for Macao Higher Education Institutions in the Area of Research in Humanities and Social Sciences and Specialized Subsidy Scheme for Prevention and Response to Major Infectious Diseases (No. HSS-MUST-2020-9).

## Conflict of Interest

The authors declare that the research was conducted in the absence of any commercial or financial relationships that could be construed as a potential conflict of interest.

## Publisher's Note

All claims expressed in this article are solely those of the authors and do not necessarily represent those of their affiliated organizations, or those of the publisher, the editors and the reviewers. Any product that may be evaluated in this article, or claim that may be made by its manufacturer, is not guaranteed or endorsed by the publisher.

## References

[1] Chau JPC Lo SHS Saran R Leung CHY Lam SKY Thompson DR. Nurses' experiences of caring for people with COVID-19 in Hong Kong: a qualitative enquiry. BMJ Open. (2021) 11:e052683. 10.1136/bmjopen-2021-052683 34426473 PMC8384498

[2] Jabbour R Harakeh M Sailan SD Nassar V Tashjian H Massouh J. Nurses' stories from Beirut: The 2020 explosive disaster on top of a pandemic and economic crises. Int Nurs Rev. (2021) 68:1–8. 10.1111/inr.12675 33891770 PMC8250565

[3] Catton H. COVID-19: The future of nursing will determine the fate of our health services. Int Nurs Rev. (2021) 68:9–11. 10.1111/inr.12673 33891771 PMC8250598

[4] Organization WH. State of the World's Nursing Report - 2020. Available online at: https://www.who.int/publications/i/item/9789240003279 (accessed October 25, 2021).

[5] Goodin HJ. The nursing shortage in the United States of America: an integrative review of the literature. J Adv Nurs. (2003) 43:335–43. 10.1046/j.1365-2648.2003.02722_1.x 12887349

[6] Leung PPL Wu CH Kwong CK Ching WK. Nursing shortage in the public healthcare system: an exploratory study of Hong Kong. Enterp Inf Syst. (2020) 14:913–31. 10.1080/17517575.2019.1569264

[7] Alsubaie A Isouard G. Job satisfaction and retention of nursing staff in Saudi hospitals. Asia Pac J Health Manag. (2019) 14:68–73. 10.24083/apjhm.v14i2.215

[8] Lee J. Nursing home nurses' turnover intention: a systematic review. Nursing Open. (2022) 9:22–9. 10.1002/nop2.1051 34811952 PMC8685779

[9] Mahoney CB Lea J Schumann PL Jillson IA. Turnover, burnout, and job satisfaction of certified registered nurse anesthetists in the United States: role of job characteristics and personality. AANA J. (2020) 88:39–48. 32008617

[10] Li N Zhang L Xiao G Chen ZJ Lu Q. Effects of organizational commitment, job satisfaction and workplace violence on turnover intention of emergency nurses: a cross-sectional study. Int J Nurs Pract. (2020) 26:e12854. 10.1111/ijn.12854 32529786

[11] Qian Y Wang X Fang X Li L. Relationship of psychological capital, job satisfaction, and retention willingness of nurses. J Shanghai Jiaotong Univ Med Sci. (2015) 35:887–91.

[12] Atefi N Abdullah KL Wong LP Mazlom R. Factors influencing job satisfaction among registered nurses: a questionnaire survey in Mashhad, Iran. J Nurs Manag. (2015) 23:448–58. 10.1111/jonm.12151 24102706

[13] Han K Trinkoff AM Gurses AP. Work-related factors, job satisfaction and intent to leave the current job among United States nurses. J Clin Nurs. (2015) 24:3224–32. 10.1111/jocn.12987 26417730

[14] Smith HL Hood JN Waldman JD Smith VL. Creating a favorable practice environment for nurses. J Nurs Admin. (2005) 35:525–32. 10.1097/00005110-200512000-00006 16344646

[15] Cortese CG Colombo L Ghislieri C. Determinants of nurses' job satisfaction: the role of work-family conflict, job demand, emotional charge and social support. J Nurs Manag. (2010) 18:35–43. 10.1111/j.1365-2834.2009.01064.x 20465727

[16] Gottlieb LN Gottlieb B Bitzas V. Creating empowering conditions for nurses with workplace autonomy and agency: how healthcare leaders could be guided by strengths-based nursing and healthcare leadership (SBNH-L). J Healthcare Leader. (2021) 13:169–81. 10.2147/JHL.S221141 34349581 PMC8326221

[17] Ho LH Chang SC Kau K Shiu SY Huang SS Wang YJ. The impact of organizational support on practice outcomes in nurse practitioners in Taiwan. J Nurs Res. (2021) 29:e148. 10.1097/JNR.0000000000000425 33756519 PMC8126501

[18] Dutra CKdR Guirardello EdB. Nurse work environment and its impact on reasons for missed care, safety climate, and job satisfaction: a cross-sectional study. J Adv Nurs. (2021) 77:2398–406. 10.1111/jan.14764 33565146

[19] Hudgins T Brown KD Layne D Stephens TM. The effect of academic nurse leaders' toxic behaviors. J Nurs Edu. (2022) 61:88. 10.3928/01484834-20211213-02 35112950

[20] Mueller CW McCloskey JC. Nurses job-satisfaction. - a proposed measure. Nurs Res. (1990) 39:113–7. 10.1097/00006199-199003000-00014 2315065

[21] Ellenbecker CH Byleckie JJ. Home healthcare nurses' job satisfaction scale: Refinement and psychometric testing. J Adv Nurs. (2005) 52:70–8. 10.1111/j.1365-2648.2005.03556.x 16149983

[22] Lu H While AE Barriball KL. Job satisfaction among nurses: a literature review. Int J Nurs Stud. (2005) 42:211–27. 10.1016/j.ijnurstu.2004.09.003 15680619

[23] Kaya A Dalgic AI. Examination of job satisfaction and burnout status of pediatric nurses: a cross-sectional and correlational study using online survey research in Turkey. Perspect Psychiatr Care. (2021) 57:800–8. 10.1111/ppc.12617 32924165

[24] Serafin L Bjersa K Doboszynska A. Nurse job satisfaction at a surgical ward. - a comparative study between Sweden and Poland. Med Pr. (2019) 70:155–67. 10.13075/mp.5893.00768 30816883

[25] Yasin YM Kerr MS Wong CA Belanger CH. Factors affecting job satisfaction among acute care nurses working in rural and urban settings. J Adv Nurs. (2020) 76:2359–68. 10.1111/jan.14449 32542730

[26] Lu H Barriball KL Zhang X While AE. Job satisfaction among hospital nurses revisited: A systematic review. Int J Nurs Stud. (2012) 49:1017–38. 10.1016/j.ijnurstu.2011.11.009 22189097

[27] Dalic I Ateljevic J Stevic Z Terzic S. An integrated swot. - fuzzy piprecia model for analysis of competitiveness in order to improve logistics performances. Facta Universitatis Ser. Mech Eng. (2020) 18:439–51. 10.22190/FUME200325029D

[28] Lizarralde R Ganzarain J Zubizarreta M. Adaptation of the MIVES method for the strategic selection of new technologies at an R&D centre. Focus on the manufacturing sector. Technovation. (2022) 115:102462. 10.1016/j.technovation.2022.102462

[29] Rouyendegh BD Savalan Ş. An integrated fuzzy MCDM hybrid methodology to analyze agricultural production. Sustainability. (2022) 14:4835. 10.3390/su14084835

[30] Alossta A Elmansouri O Badi I. Resolving a location selection problem by means of an integrated AHP-RAFSI approach. Int J Inf Manage. (2021) 2:135–42. 10.31181/rme200102135a

[31] Hsu WCJ Liou JJH Lo H-W. A group decision-making approach for exploring trends in the development of the healthcare industry in Taiwan. Decision Support Systems. (2021) 141:113447. 10.1016/j.dss.2020.113447

[32] Biswas S. Measuring performance of healthcare supply chains in India: A comparative analysis of multi-criteria decision making methods. Decis Mak Appl Manag Eng. (2020) 3:162–89. 10.31181/dmame2003162b

[33] Mardani A Hooker RE Ozkul S Sun YS Nilashi M Sabzi HZ. Application of decision making and fuzzy sets theory to evaluate the healthcare and medical problems: A review of three decades of research with recent developments. Expert Syst Appl. (2019) 137:202–31. 10.1016/j.eswa.2019.07.002

[34] Bonyadi N Asghari H Kiaei M. Identification and Prioritization of Employee Satisfaction Strategies in Tehran Regional Water Company Using Analytic Hierarchy Process (AHP). Tehnicki Glasnik-Tech J. (2020) 14:251–6. 10.31803/tg-20191129112717

[35] Tsai SB. Using the DEMATEL model to explore the job satisfaction of research and development professionals in china's photovoltaic cell industry. Renew Sustain Energy Rev. (2018) 81:62–8. 10.1016/j.rser.2017.07.014

[36] Xin L Ting H Chunrong L. Kalra J Lightner NJ Taiar R, editors. Job Satisfaction and Expectations of Pharmacy Employees During the COVID-19 Pandemic: An Application of DEMATEL Method. Geneva: WHO (2021). p. 667–74.

[37] Joanne M. Influence of rewards and incentives on staff nurse turnover rate. Nurs Res. (1974) 23:239–47. 10.1097/00006199-197405000-00009 4494858

[38] Wilkinson CS Hite KJ. Nurse-physician collaborative relationship on nurses' self-perceived job satisfaction in ambulatory care. Lippincotts Case Manag. (2001) 6:68–78. 10.1097/00129234-200103000-00005 16398009

[39] Lynch SA. Job satisfaction of home health nurses. Home Healthc Nurse. (1994) 12:21–8. 10.1097/00004045-199409000-00004 7960880

[40] Robertson EM Higgins L Rozmus C Robinson JP. Association between continuing education and job satisfaction of nurses employed in long-term care facilities. J Contin Educ Nurs. (1999) 30:108–13. 10.3928/0022-0124-19990501-06 10640068

[41] Abu Ajamieh AR Misener T Haddock KS Gleaton JU. Job satisfaction correlates among Palestinian nurses in the West Bank. Int J Nurs Stud. (1996) 33:422–32. 10.1016/0020-7489(95)00068-2 8836766

[42] Al-Enezi N Chowdhury RI Shah MA Al-Otabi M. Job satisfaction of nurses with multicultural backgrounds: a questionnaire survey in Kuwait. Appl Nurs Res. (2009) 22:94–100. 10.1016/j.apnr.2007.05.005 19427570

[43] Tourangeau AE Hall LM Doran DM Petch T. Measurement of nurse job satisfaction using the McCloskey/Mueller Satisfaction Scale. Nurs Res. (2006) 55:128–36. 10.1097/00006199-200603000-00008 16601625

[44] Clinton M Dumit NY El-Jardali F. Rasch measurement analysis of a 25-item version of the Mueller/McCloskey nurse job satisfaction scale in a sample of nurses in Lebanon and Qatar. Sage Open. (2015) 5:1–10. 10.1177/2158244015592167

[45] Gabus A Fontela E. World Problems, An Invitation to Further Thought Within the Framework of DEMATEL. Geneva: Battelle Institute, Geneva Research Center (1972).

[46] Kamble SS Gunasekaran A Sharma R. Modeling the blockchain enabled traceability in agriculture supply chain. Int J Inform Manag. (2020) 52:101967. 10.1016/j.ijinfomgt.2019.05.023

[47] Huang CN Lo HW. A Hybrid Z-Based MADM model for the evaluation of urban resilience. Math Prob Eng. (2021) 2021:1–17. 10.1155/2021/9474753

[48] Badri Ahmadi H Lo H-W Gupta H Kusi-Sarpong S Liou JJH. Analyzing interrelationships among environmental sustainability innovation factors. Clean Technol Environ Policy. (2022) 24:1191–207. 10.1007/s10098-021-02086-z

[49] Maqbool A Khan NZ. Analyzing barriers for implementation of public health and social measures to prevent the transmission of COVID-19 disease using DEMATEL method. Diabetes Metab Syndr. (2020) 14:887–92. 10.1016/j.dsx.2020.06.024 32563940 PMC7293847

[50] Saaty TL. The Analytic Hierarchy Process : Planning, Priority Setting, Resource Allocation. McGraw-Hill (1980).

[51] Rezaei J. Best-worst multi-criteria decision-making method. Omega. (2015) 53:49–57. 10.1016/j.omega.2014.11.009

[52] Si S-L You X-Y Liu H-C Zhang P. DEMATEL technique: a systematic review of the state-of-the-art literature on methodologies and applications. Math Prob Eng. (2018) 2018:3696457. 10.1155/2018/3696457

[53] Garg CP. Modeling the e-waste mitigation strategies using grey-theory and DEMATEL framework. J Clean Prod. (2021) 281:124035. 10.1016/j.jclepro.2020.124035

[54] Saaty T. Decision Making with Dependence and Feedback: The Analytic Network Process. International. Rws publications (1996). p. 25–44.

[55] Liou JJH Chuang YC Zavadskas EK Tzeng GH. Data-driven hybrid multiple attribute decision-making model for green supplier evaluation and performance improvement. J Clean Prod. (2019) 241:118321. 10.1016/j.jclepro.2019.118321

[56] Chuang YC Tung TH Chen JY Chien CW Shen KY. Exploration of the relationship among key risk factors of acute kidney injury for elderly patients considering Covid-19. Front Med. (2021) 8:639250. 10.3389/fmed.2021.639250 34368176 PMC8339321

[57] Martilla JA James JC. Importance-performance analysis. J Mark. (1977). 10.2307/1250495

[58] Tsai SB Huang CY Wang CK Chen Q Pan J Wang G. Using a mixed model to evaluate job satisfaction in high-tech industries. PLoS ONE. (2016) 11:e0154071. 10.1371/journal.pone.0154071 27139697 PMC4854457

[59] Ishak A Asfriyati Noviza MB editors. Analysis of employees satisfaction index to management of transportation facilities office using importance performance analysis (IPA): case study. In: 1st International Conference on Industrial and Manufacturing Engineering (ICI and ME). Medan: Univ Sumatera Utara, Master & Doct Program Ind Engn (2019).

[60] Ford JB Joseph M Joseph B. Importance-performance analysis as a strategic tool for service marketers: The case of service quality perceptions of business students in New Zealand and the USA. J Serv Mark. (2005) 13:171–86. 10.1108/08876049910266068

[61] Hosseini SY Bideh AZ. A data mining approach for segmentation-based importance-performance analysis (SOM-BPNN-IPA): a new framework for developing customer retention strategies. Service Business. (2014) 8:295–312. 10.1007/s11628-013-0197-7

[62] Ainin S Hisham NH. Applying importance-performance analysis to information systems: an exploratory case study. J Inform Inform Technol Organ. (2008) 3:95–103. 10.28945/132

[63] Chu RKS Choi T. An importance-performance analysis of hotel selection factors in the Hong Kong hotel industry: a comparison of business and leisure travellers. Tour Manag. (2000) 21:363–77. 10.1016/S0261-5177(99)00070-9

[64] Ravangard R Yasami S Shokrpour N Sajjadnia Z Farhadi P. The effects of supervisors' support and mediating factors on the nurses' job performance using structural equation modeling: a case study. Health Care Manag. (2015) 34:265–76. 10.1097/HCM.0000000000000068 26218002

[65] McHugh MD Kutney-Lee A Cimiotti JP Sloane DM Aiken LH. Nurses' widespread job dissatisfaction, burnout, and frustration with health benefits signal problems for patient care. Health Aff. (2011) 30:202–10. 10.1377/hlthaff.2010.0100 21289340 PMC3201822

[66] Abualrub RF Omari FH Abu Al Rub AF. The moderating effect of social support on the stress-satisfaction relationship among Jordanian hospital nurses. J Nurs Manag. (2009) 17:870–8. 10.1111/j.1365-2834.2009.01007.x 19793244

[67] Lopez-Ibort N Canete-Lairla MA Gil-Lacruz AI Gil-Lacruz M Antonanzas-Lombarte T. The quality of the supervisor-nurse relationship and its influence on nurses' job satisfaction. Healthcare. (2021) 9:1388. 10.3390/healthcare9101388 34683067 PMC8544584

[68] Holmberg C Sobis I Carlstrom E. Job satisfaction among Swedish mental health nursing staff: a cross-sectional survey. Int J Public Admin. (2016) 39:429–36. 10.1080/01900692.2015.101843218352967

[69] Bhandari KKT Xiao LD Belan I. Job satisfaction of overseas-qualified nurses working in Australian hospitals. Int Nurs Rev. (2015) 62:64–74. 10.1111/inr.12146 25418010

[70] Bellizzi S Padrini S. Report of the “satisfaction” survey amongst public health services nurses in port said. Bmc Nursing. (2021) 20:1–5. 10.1186/s12912-021-00707-y 34649536 PMC8518242

[71] Zhang WJ. Dimission reasons of contract nurses in the public hospitals from the perspective of labor relations. Chin Nurs Manag. (2012). 12:44–6. 10.3969/j.issn.1672-1756.2012.09.013

[72] Misfeldt R Linder J Lait J Hepp S Armitage G Jackson K. Incentives for improving human resource outcomes in health care: overview of reviews. J Health Serv Res Policy. (2014) 19:52–61. 10.1177/1355819613505746 24170147

[73] Spetz J Herrera C. Changes in nurse satisfaction in California, 2004 to 2008. J Nurs Manag. (2010) 18:564–72. 10.1111/j.1365-2834.2010.01117.x 20636505

[74] Bimpong KAA Khan A Slight R Tolley CL Slight SP. Relationship between labour force satisfaction, wages and retention within the UK national health service: a systematic review of the literature. BMJ Open. (2020) 10:e034919-e. 10.1136/bmjopen-2019-034919 32699127 PMC7375434

[75] Pittman J. Registered nurse job satisfaction and collective bargaining unit membership status. J Nurs Admin. (2007) 37:471–6. 10.1097/01.NNA.0000285148.87612.72 17914295

[76] Kim MH Yi YJ. Impact of leader-member-exchange and team-member-exchange on nurses' job satisfaction and turnover intention. Int Nurs Rev. (2019) 66:242–9. 10.1111/inr.12491 30474113

[77] Gözde Koca SY. Bibliometric analysis of DEMATEL method. Decis Mak Appl Manag Eng. (2021) 4:81–103. 10.31181/dmame2104085g

[78] López-Ospina H Pardo D Rojas A Barros-Castro R Palacio K Quezada L. revisited fuzzy DEMATEL and optimization method for strategy map design under the BSC framework: selection of objectives and relationships. Soft Comput. (2022) 26:6619–44. 10.1007/s00500-022-07042-7

[79] Du Y-W Li X-X. Hierarchical DEMATEL method for complex systems. Expert Syst Appl. (2021) 167:113871. 10.1016/j.eswa.2020.11387134270630

